# Supplementary material for: Wombs for rent: Exploring the motivations behind Ghanaian Women’s decisions to become surrogate mothers
Source: PLoS One. 2026 Apr 7;21(4):e0346006. doi: 10.1371/journal.pone.0346006 (PMC13056194; doi:10.1371/journal.pone.0346006)
Supplement: S1 File — (DOCX) [file pone.0346006.s001.docx]

| **Motivations for Surrogacy: Coding Scheme** | | |
| --- | --- | --- |
| **Category** | **Description** | **Examples** |
| Financial motivation | pg. 1Participants indicated that financial gain was the primary factor driving their decision to participate in surrogacy. | “I needed money terribly to pay for my school fees and take care of my children; there was nowhere to turn to so I accepted the offer to be a surrogate mother when a friend told me about it”.  “Madam at that point I needed to get the money to pay for the accommodation, the landlord was chasing us for the rent advance and the financial situation was very bad for me and my husband so when I discussed it with one of my friends, she then told me that there is something like surrogacy which I can do and get money so I decided to do it”  “For me, it was because of the money that I went in for it. It’s not easy carrying a pregnancy for nine months and giving birth for someone, but I needed the money. That’s why I did it. My friend told me about surrogacy when I asked her if she knew someone who could lend me money to expand my provision shop, which was struggling. I agreed because I wouldn’t have any debt afterward”.  “At that point, I desperately needed money to take care of my children. I was unemployed and had to pay my children’s school fees and start a small business. I had no other choice.”  “The truth is, I wanted money to start my own business and stop working at the waakye joint, where the pay was poor. When a friend told me about surrogacy, I thought it over and decided to go for it”. |
| Altruistic motivation | Some participants explicitly stated that their primary motivation for becoming surrogates was driven by altruism and/or empathy. Getting into surrogacy was driven by the need to help others. | “It’s not the money per se, even though life wasn’t easy for me so the money too is something I needed to support myself and my children but when I see someone who can’t give birth it worries me. For instance, if I can get pregnant and later want to abort the baby when someone is also looking for a baby, and I can be taken through treatment so that I can carry the baby and give it to that person, I feel it is better and it will bring blessings to me in the future but it wasn’t solely because of the money that I agreed do it.”  “Hmmmmmm, my nurse friend told me about a woman they had who needed a surrogate and that the doctors had tried to do the insemination for her several times so that she can carry her own baby but it has always failed like 4 times. I felt sorry for her and decided to help because I have 2 of my own so if I can help someone get one why not. You can imagine what she might be going through in her marriage because she doesn’t have a child”  ‘As for the money it is a secondary issue because the money they gave me was not worth the work I did as a surrogate. I will say the main thing that motivated me was the sympathy for the woman to also have a child else I wouldn’t have done it.’ |
| Religious motivation | It is found in the responses of the participants that they believe in the doctrine of offering help to others in need as proposed by their various Christian religious denominations. They in essence linked their services as surrogates as compliant with their religious beliefs about being a good person. | “The bible says, love your neighbour as yourself and I have brought a human being to this world for someone to be happy. It’s not because of money even though I was going through a lot financially when I agreed to be a surrogate, I think God will bless me for helping some couple get a child of their own”.  “As a Christian, I believe in doing good; the bible teaches us to do good. That was what motivated me to be a surrogate mother for them, not because of money though I was going through financial struggles but I wouldn’t use that means to make money.” (Irene, 27 yr. old waitress) |
|  |  |  |
