# Supplementary material for: Wombs for rent: Exploring the motivations behind Ghanaian Women’s decisions to become surrogate mothers
Source: PLoS One. 2026 Apr 7;21(4):e0346006. doi: 10.1371/journal.pone.0346006 (PMC13056194; doi:10.1371/journal.pone.0346006)
Supplement: S2 File — (DOCX) [file pone.0346006.s002.docx]

## SEMI-STRUCTURED INTERVIEW GUIDE

Study Title: The practice of surrogacy: Exploring the Lived Experiences of Gestational Surrogates in Accra

Principal investigator: Doris Ayeley Amarteifio

1. **Background information**

Code number…………………….

1. Age…………………………………………

2. Marital status…………………………………..

4. Residence………………………………………….

5. Nationality…………………………………………

6. Tribe ……………………………………………

7. Languages spoken ……………………………………….

8. Occupation ………………………………………………….

9. Religion ……………………………………………………..

10. Educational background ………………………………………

11. Number of children ……………………………………………

**B. Guiding Questions**

1. Please tell me about the day you heard of IVF surrogacy.
2. How did you receive the information?
3. What inspired you to become a surrogate mother? (probe further on her income before becoming a surrogate)
4. I would like to know about how you were engaged.
5. What happened next?
6. Tell me more about the information given on the processes involved in IVF surrogacy
7. I would like to know if you have provided any surrogate services before this and if so, how many times?
8. If you have provided any surrogate services before this one what was the mode of delivery of the baby?
9. What is your expectation on the mode of delivery of this current pregnancy?
10. Please tell me about the reason for the expectation of the mode of delivery.
11. Tell me your socio-cultural beliefs and practices in relation to your decision regarding surrogacy services.
12. How does your religion view surrogacy? Probe further
13. Tell me about your family’s reaction towards you providing surrogacy services
14. Are other close relatives and friends aware that you are providing surrogate services? Probe (does she have a partner, children)
15. If yes, what were their reactions toward the decision? If no how do you intend answering questions on the outcome of the pregnancy?
16. Where are you residing now? (probe. Has she relocated or not?)
17. What were you offered in exchange for surrogacy services?
18. What did you receive so far?
19. Did you sign any agreement with the agency before starting?
20. If yes, were you given a copy?
21. Has the agency fulfilled their side of the agreement so far? Please explain.
22. What were the benefits you perceived in providing the surrogacy service?
23. What will you say are some challenges in connection with the surrogacy service you provided?
24. What is your opinion about the treatment you received in the hospital?
25. What support have you received from the management and staff of the hospital?
26. Is there anything you think can be done to improve surrogacy services in Ghana?
27. Is there anything else you would like to share with me
