# Supplementary material for: Wombs for rent: Exploring the motivations behind Ghanaian Women’s decisions to become surrogate mothers
Source: PLoS One. 2026 Apr 7;21(4):e0346006. doi: 10.1371/journal.pone.0346006 (PMC13056194; doi:10.1371/journal.pone.0346006)
Supplement: Attachment — Submitted filename: Reviewers Comment.pdf [file pone.0346006.s003.pdf]

Comments from Reviewers on article titled, “Wombs for Rent: Exploring the Motivations behind Ghanaian Women's Decisions to Become Surrogate Mothers" Original Submission

Reviewer # 1

An interesting paper. Many comments are points for clarification and issues to reflect upon.
Table 1 – It would be useful to provide the characteristics of women who undertake surrogacy.
-bold “level of education”
In the results – did culture feature as a theme? I see a bit integrated in the section on altruism but perhaps it deserves to be a separate theme.
Page 11, the content on gay fathers – are these gay couples who are biologically male? So do the females volunteer for surrogacy out of need to support the gay fathers? What kind of women offer to become surrogate mothers in developed countries? It would help to make a comparison.
Of course, there are variations between developed and developing world settings. This is why it is important to first describe the characteristics of the respondents. Did the authors conduct interviews or review literature on the general characteristics of women who participate in surrogacy? Such would further strengthen the conclusions of the manuscript.
P16, the discussion should include contrary ideas for instance, in Christianity, where surrogacy is considered unnatural.
Isn’t is possible for poor persons to have altruistic motives? One of them even mentioned that the funds provided did not measure to the service provided. Concerning the overriding motivation being economic – a question that should have been asked is “would they have volunteered without the economic incentive”? This would strengthen the discussion on motives for participation.
Ethics – were participants allowed not to answer questions that they did not wish to answer?
Data – is plural.
Table 1 – please bold the “level of education”.

Reviewer #2

I found the abstract to be captivating. It was very well written, and it captured the various reasons for surrogacy in the unique cultural environment of Ghana very well.

I would suggest citing the very first sentence citing the enhanced scrutiny of surrogacy in low and middle income countries. There are other sentences on the final paragraph of page 3 that I would suggest providing a citation for, as well.

I suggest un-bolding of the words 'traditional' and 'gestational' on page 3.

I suggest rewording the concluding sentence on page 5 to read "We were of the view that these categories of recent surrogate mothers would be able to accurately and comprehensively recall their experiences and motivations."

I would also reword the following sentence to read "More so, surrogates who were less than 16 weeks pregnant were excluded from the study because it was deemed that they did not have enough experience with antenatal care due to their early gestational age."

In discussing how participants were contacted, the authors need to provide explanation how specifically these subjects were contacted through the agencies. Was it by telephone? Email? Social media? (this does happen in studies). The sentence structure in this paragraph overall does not seem as robustly written as the Abstract/Background, perhaps due to a language barrier ?

Remove the word "See" before ("Table 1")

In the ethical considerations section, I would edit the paragraph to read "participants were appropriately counselled on the merits of their participation in this study, the confidentiality of their participation and responses was assured, and it was communicated that they could withdraw from the study at any point without consequence." The phrase "made to understand" sounds somewhat forceful. Following this, remove the capital "P" from the word "pseudonyms".

I do not believe it is necessary to note that the interviews were conducted by the "second author" or "third author" (both on page 6)…. I would instead simply replace this with "interviewer".

In data analysis, replace "over and over" with "multiple times". I would also replace those ideas

that coding was “noted”, instead of “jotted down”.

Replace “there is not much overlap” with “minimal”. Replace “put aside” with “disregarded”. Replace “the themes that remained are those that tell a story about the data in relation to the research question” with “the themes that remained captured the data from participant responses, and were pertinent to the overall premise of the study itself.”

It is troubling that the authors noted that they used Nvivo for data analysis and coding of data, but this data is not included in any way, with no thematic maps included. This information must be provided.

For the socio-economic/financial motivation paragraph, it would be helpful to provide quantification data regarding the responses. For example, it notes that for some women, financial rewards for business purposes was a motivating factor; how many subjects noted this ? How many sought financial assistance for their own education? This data should be presented in a table, and potentially specified in the text.

The discussion which follows is excellent.

I would replace the word “gay” with “homosexual” in the last paragraph on page 11.

I would reword the sentences “Thus, whilst altruism may be the primary reason, the potential to make some money could also not be taken out of the equation. In fact, the reverse could be true where financial considerations may be primary and altruism, secondary for many surrogate mothers.” With Thus, while altruism may be the primary reason, personal financial gain remains a significant secondary factor. Conversely, it is possible that financial considerations may be primary, while altruism is secondary for many surrogate mothers.”

On page 16, I would combine the paragraphs starting with “although participants expressed altruistic”, and “for many surrogates, their financial” into a single paragraph, as both are discussing financial reasons. I also would combine the two paragraphs on page 17.

Overall, this is an interesting study, much of which is very well written, and I believe it is worthy of publication. However, additional data on patient responses needs to be provided.

Reviewer #3

The study is interesting, providing information about a specific population that was not previously known, but it does not bring anything new or different from what was expected to be

found.

The sample size is small, although it is a qualitative study, the small sample size does not allow generalizations to the entire population.

The study was approved by an ethics committee, which accepted informed consent in oral form. Although it was a survey conducted by telephone, it could have been conducted in two stages, with a prior period to explain the study and request the submission of a written informed consent form.

The authors do not provide an interview script, but they should. There are very relevant aspects that are not mentioned, so we are left wondering whether they were asked, such as whether the woman already has children, and how her family reacted to the surrogacy process.

Regarding the motivations for this practice, all participants cite economic reasons as having weight in their decision. It would be important to question whether this decision would be maintained if there were no payment for it.

There are very relevant ethical issues that justify the non-acceptance of surrogacy in many countries. In others, it is permitted, but in an altruistic manner, that is, without payment for this practice. In still others, such as India (one of the few countries cited by the authors), it is no longer permitted for non-resident foreigners. It would be important to delve deeper into these issues, and there is literature available on the subject.

---- Best Wishes ---
