# Supplementary material for: Wombs for rent: Exploring the motivations behind Ghanaian Women’s decisions to become surrogate mothers
Source: PLoS One. 2026 Apr 7;21(4):e0346006. doi: 10.1371/journal.pone.0346006 (PMC13056194; doi:10.1371/journal.pone.0346006)
Supplement: Attachment — Submitted filename: womb for rent 2 reviewer 2.docx [file pone.0346006.s005.docx]

Thank you to the authors of this paper for addressing most of my previous comments. It is helpful they included the number of responses for cited motivations, though I believe this would read cleaner if it was provided in a format of, for example (n=3) instead of just (3), as the latter could be mistaken for a citation.

I also agree with the third reviewer, an interview script of the asked questions (not transcript of responses) should be provided. The explanation that further analysis and additional manuscript preparation are in process is not a sufficient reason to withhold this. If this list of questions is too long to include in the main paper, it could be included as a supplementary attachment.

I am otherwise satisfied with the revisions. I would encourage the authors to carefully view the manuscript line by line to address typos (ie, two periods after the word “motivations” on the top of page 5 are present), and inconsistencies to ensure that the paper is polished for final publication.
